# Haploid embryonic stem cells serve as a new tool for mammalian genetic study

**DOI:** 10.1186/scrt409

**Published:** 2014-02-06

**Authors:** Ling Shuai, Qi Zhou

**Affiliations:** 1 State Key Laboratory of Reproductive Biology, Institute of Zoology, Chinese Academy of Sciences, 1 Beichen West Road, Beijing, Chaoyang District 100101, PR China

## Abstract

In mammals, all somatic cells carry two sets of chromosomes while haploids are restricted only to gametes and are occasionally found in tumors with genome instability. Mammalian haploid embryonic stem (ES) cells have recently been established successfully in mice and monkeys, from either parthenogenetic or androgenetic haploid embryos. These haploid ES cells maintain haploidy and stable growth during extensive *in vitro* culture, express pluripotent markers, and possess the ability to differentiate into all three germ layers *in vitro* and *in vivo*. The mouse haploid ES cells can also contribute to germlines of chimeras. Moreover, the mouse androgenetic haploid ES cells can produce fertile progenies after intracytoplasmic injection into mature oocytes, and the mouse parthenogenetic haploid ES cells can also achieve this by substitution of the maternal genome, albeit at a lower efficiency. These distinct features of mammalian haploid ES cells empower themselves not only as a valuable tool for genetic screening at a cellular level, but also as a new tool for genome-modified animal production and genetic studies at the animal level. Here we review the current progress on mammalian haploid ES cell research, describe in detail their characteristics, and discuss their potential applications. These achievements may provide a new but powerful tool for mammalian genetic studies, and may also shed light on the some interesting questions regarding genome ploidy maintenance and genomic imprinting.

## Introduction

Haploid cells are optimal tools for genetic screening due to their single-set chromosome feature. Compared with using diploid cells, it is much easier to use haploid cells to produce homozygous mutants that are essential for study of recessive traits. Haploid cells only exist naturally in yeast and bees [1] and some types of plants [2], which have been widely used in recessive phenotype analysis. However, haploids in mammals are only restricted to gametes, which can hardly survive *in vitro* for long-term culture to fulfill genetic manipulations. Establishing mammalian haploid cell lines for genetic screening and to study recessive traits in mammals would therefore be of great value.

Since the 1970s, large efforts have been made to generate haploid embryos in the mouse, such as derivation of haploid mouse embryos by different strategies [3-5]. The successful establishment of mouse embryonic stem (ES) cells shed light on haploid cell derivation in the mouse [6], which raised the chances of establishing haploid mouse ES cells from haploid mouse embryos. Although haploid embryos could be generated from parthenogenetic activated metaphase II oocytes, no haploid ES cell lines were derived due to their rapid auto-diploidization during cell culture *in vitro*[7]. In the past three decades, generation of mammalian haploid cell lines has not been achieved, limiting mammalian genetic screening. Recently, near-haploid cells were derived from human tumor cells [8,9], and were used in genetic screening as an efficient and convenient method. However, they were not yet authentic haploid cell lines due to the genome instability.

The first successful derivation of vertebrate haploid cells came in 2009 with the establishment of haploid ES cells in Medaka fish, which not only maintained haploidy during cell culture *in vitro* but also held pluripotency like conventional ES cells [10]. After that, several breakthroughs were accomplished in mammals, including the derivation of two types (androgenetic origin and parthenogenetic origin) of mouse haploid ES cells, and the monkey parthenogenetic haploid ES (phES) cells. These unique mammalian haploid cells can maintain haploidy and genome integrity very well after extensive culture *in vitro*, and hold pluripotency as revealed by the expression of pluripotent markers and differentiation into three germ layers including the germline. Importantly, the mammalian haploid ES cells excelled themselves not only as efficient genetic screens at a cellular level, but also in the production of gene-modified animals by substituting the gamete genome at the animal level.

Here, we review the characteristics of mammalian haploid ES cells with a focus on their pluripotency and epigenetic features, and further discuss the potential application of these cells in recessive gene screens and transgenic animal model production.

## Establishment of haploid embryonic stem cells

Mouse haploid embryos could be generated by micromanipulation of fertilized eggs [3] or by parthenogenetic activation of metaphase II oocytes [4]. Kaufman and colleagues tried to establish haploid ES cell lines from mouse haploid embryos, but failed due to the lack of a suitable method to maintain the haploidy of these cells [7]. Until recently, fish haploid ES cells were successfully established, indicating that haploid cells could be established in a pluripotent state [10,11]. However, fish haploid ES cells could maintain their haploid state well even when cultured *in vitro*, with no auto-diploidization. But mammalian haploid ES cells were weaker in a haploid state than fish haploid ES cells (Table 1), and therefore how to obtain mammalian haploid ES cells was still unsolved.

**Table 1:** Summary of vertebrate haploid cells

**Cell type**	**Origin**	**Pluripotency**	**Germline competence**	**Dependent factors for self-renewal**	**Genome stability**	**Function as gametes**
Fish phES cells	Parthenogenetic haploid embryos	Yes	Yes	bFGF and LIF	Stable	Yes
Mouse phES cells	Parthenogenetic haploid embryos	Yes	Yes	2i and LIF	Stable, with auto-diploidization	Yes
Mouse ahES cells	Androgenetic haploid embryos	Yes	Yes	2i and LIF	Stable, with auto-diploidization	Yes
Monkey phES cells	Parthenogenetic haploid embryos	Yes	ND	bFGF	Stable, with auto-diploidization	ND
Human near-haploid cells	Tumors	No	No	ND	Unstable, with chromosome loss	No

*ahES*, androgenetic haploid embryonic stem; *bFGF*, basic fibroblast growth factor; *LIF*, leukemia inhibitory factor; *ND*, not determined; *phES*, parthenogenetic haploid embryonic stem.

In 2011, Leeb and Wutz [12] and Elling and colleagues [13] reported independently that mouse phES cells could be derived from parthenogenetic haploid embryos via Hoechst 33342 staining and fluorescence-activated cell sorting (FACS) of haploid cells. The methods for generation of haploid embryos and derivation of ES cells were similar to previous reports, with slight modifications. They introduced 2i into their phES cell derivation protocol to promote the ground state of phES cells [14], and sorted haploid cells via FACS according to their DNA contents by Hoechst 33342 staining. Based on this novel haploid cell enrichment method, mouse haploid ES cells could be maintained in long-term culture *in vitro*. Evidence showed that mouse haploid ES cells shared typical ES cell features with conventional ES cells, such as self-renewal and various differentiation potentials. The mouse haploid ES cells could therefore be applied widely in gene targeting or RNA interference to track the recessive phenotype in the mouse.

**Figure 1:**
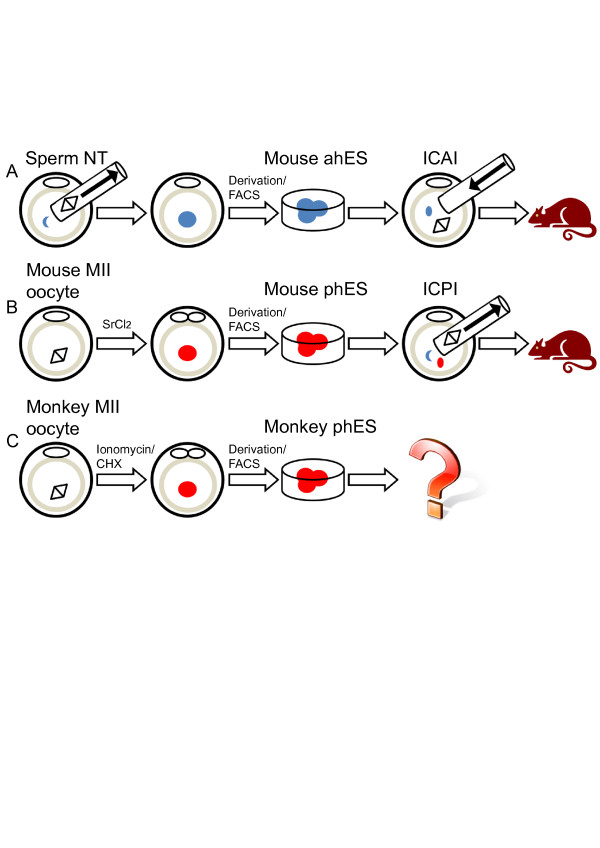
**Derivation of mammalian haploid embryonic stem cells and their development potentials. (A)** Reconstruction of mouse androgenetic haploid embryos by the sperm nuclear transfer (NT) process. Mouse androgenetic haploid embryonic stem (ahES) cells could be derived from these embryos via embryonic stem cell derivation and fluorescence-activated cell sorting (FACS) enrichment. Fertile mice could then be obtained by the intracytoplasmic ahES cell injection (ICAI) procedure. **(B)** Derivation of mouse parthenogenetic haploid embryonic stem (phES) cells and production of fertile mice by substituting maternal genomes of zygotes with phES cells. **(C)** Derivation of monkey phES cells. Whether the monkey phES could support embryonic development via the intracytoplasmic phES cell injection (ICPI) process is not yet clear. CHX, cycloheximide; MII, metaphase II.

In 2012, Yang and colleagues [15] and Li and colleagues [16] reported independently that mouse androgenetic haploid ES (ahES) cells could be established from reconstructed androgenetic haploid embryos. Yang and colleagues recently reported the successful derivation of monkey phES cells from parthenogenetic activated haploid embryos [17]. The schematic overview for derivation of the three types of mammalian haploid ES cells is shown in Figure 1. Unlike mouse phES cells, monkey phES cells seemed very stable in maintaining a haploid state. The mechanisms underlying the haploidy maintenance for mice and monkeys are still unclear (Table 1).

## Pluripotency of haploid embryonic stem cells

To date, all reported mouse haploid ES cells contain 19 + X chromosomes, and none has 19 + Y chromosomes. This observation is consistent with haploid embryos without the X chromosome (19 + Y set) presenting defects in pre-implantation development [18]. According to comparative genomic hybridization analysis or deep sequencing, haploid ES cells displayed few amplifications or deletions, indicating that these cells could maintain genome integrity during long-term culture *in vitro* (Table 1). Besides, mouse haploid ES cells showed standard domed-like mouse ES cell morphology, and expressed pluripotent markers (for example, Oct4, Nanog, Sox2). Similar to diploid ES cells, mouse haploid ES cells could form teratomas (containing cell types of three germ layers) in severe combined immune deficiency mice. Using an *in vivo* development assay, mouse haploid ES cells could produce chimeric mice and could contribute to the germline [19].

To assess whether the haploid state could be maintained during haploid ES cell differentiation, chimeric embryos were produced from ahES cells carrying green fluorescence protein and the DNA contents of green fluorescence protein-labeled cells were analyzed. Only on embryonic day 6.5 did the dissociated green fluorescence protein cells have a small population of haploid cells. However, in embryos of later stages, such as embryonic days 8.5 and 12.5, all green fluorescence protein-labeled cells were diploidized, suggesting that haploid ES cells underwent rapid diploidization during their differentiation process. Consistent with the *in vivo* experiment, Li and colleagues derived epiblast-like haploid stem cells from ahES cells by differentiation *in vitro*, which showed many features of primed-state stem cells and suggested that haploid stem cell lines could be established beyond the naïve ES cell state [16]. However, during further differentiation of these epiblast-like haploid stem cells into neural cells, only a few haploid Nestin-positive cells could be detected at the very beginning of neural differentiation, and no haploid cells could be obtained in further differentiated stages such as mouse haploid ES cells. Monkey phES cells also expressed pluripotent markers, and could form embryonic bodies *in vitro* and teratoma *in vivo* containing three germ layers. Taken together, both mouse and monkey haploid ES cells could self-renew with an intact haploid genome and a pluripotent state (Table 1).

## Production of fertile mice from haploid embryonic stem cells

Considering the gamete origin of haploid ES cells, they may maintain parent-specific genome imprints that are essential for normal development, raising the potential to use haploid ES cells to function as gametes supporting embryonic development. Yang and colleagues [15] and Li and colleagues [16] proved this hypothesis independently by intracytoplasmic ahES cell injection (ICAI) into oocytes, and both groups generated fertile mice efficiently with ahES cells. After injection into metaphase II oocytes and chemical (SrCl_2_) stimulus activation, the genome of the donor cell undergoes a fast demethylation process accompanied with reprogramming. Around 5 to 6 hours later, these reconstructed embryos would form a paternal pseudo-pronucleus and a maternal pronucleus, which were quite similar to the fertilized zygotes. Full-term pups could be generated by transferring these embryos back to the uterus of pseudo-pregnant mice. Most of the pups could develop to adulthood and give birth to the next generation normally, but some newborn pups generated by ICAI assay died shortly after birth because of developmental retardation, which might be due to an abnormal imprinting state in donor ahES cells. Through loss of imprinting in some imprinted genes during long-term culture in diploid ES cells [20], a similar phenomenon was also found in ahES cells. It would be a useful strategy to modify the imprint status of ahES cells for efficient generation of ICAI mice in the future.

Equally, the phES cells that inherit oocyte genomes could support development by substituting maternal genomes of fertilized zygotes. Wan and colleagues proved this through a proof-of-principle experiment: first, they injected one sperm head into an enucleated oocyte; second, they performed intracytoplasmic phES cell injection; third, they activated these reconstructed embryos by chemical stimulus (SrCl_2_) and transferred them back to pseudo-pregnant mice; and, finally, live mice were produced by this process and could give birth to the next generation, although the efficiency was low [21]. The lower efficiency of the intracytoplasmic phES cell injection procedure may be caused by two reasons: the loss of imprinting in phES cells, or the enucleation manipulation that is very harmful to the development of reconstructed embryos.

Although the method of production of animals via ahES cells and phES cells needs to be optimized to improve the efficiency, it does offer a new approach to produce gene-manipulated animal models that is distinct from traditional methods based on germline competent ES cells.

## Application of haploid embryonic stem cells in genetic screens

Haploid ES cells very easily generate homozygous mutants, and the double haploids are also homozygous. This makes haploid ES cells a valuable tool for genetic screens. Further, the screened mutant haploid ES cells could transmit gene modification to an animal level by the ICAI or the intracytoplasmic phES cell injection procedure, which provides a convenient way to produce a transgenic animal model.

Elling and colleagues proved the benefits of using haploid ES cells for genetic screens by simply transfecting cells with retrovirus, and they easily gained millions of insertional mutations covering the genome in a single experiment [13]. They also performed a forward screen with the potential bioweapon ricin in mutant phES cells, and easily obtained resistant cell lines. According to the analysis, they found that Gpr107 might mediate ricin toxicity. Yang and colleagues proved that homologous recombination could be achieved in ahES cells to fulfill gene targeting, a powerful tool for reverse genetic studies [15]. Li and colleagues performed random transgenesis of the *neo*-resistant gene in ahES cells [16], and showed that the transgenesis efficiency was similar between haploid and diploid ES cells and that the transgenic manipulation did not compromise the developmental efficiency of ahES cells in the ICAI procedure, raising the possibility of producing gene-modified mice with recent gene manipulation technologies (transposon mutagenesis [22], zinc-finger nuclease [23], TALEN-mediated gene targeting [24], or CRISPR/Cas-mediated genome engineering [25]).

Recent remarkable work showed that reversible gene trap collection was generated in the human near-haploid cell line KBM7, which empowered genetic screening in humans [26]. This study provides a platform to produce a library carrying many single gene-trap insertions that will serve in cellular phenotype screens, and suggests the importance of generating human and other types of mammalian haploid ES cell lines for genetic studies.

## Perspectives

Haploid ES cells have made great progress in mammalian genetic studies. They serve as a valuable tool not only at a cellular level in genetic screens but also at an animal level in transgenic animal production compared with conventional diploid ES cells. However, before being widely used in gene function research and drug selection, there are still some problems that need to be solved in haploid ES cells. The mechanism of genome ploidy maintenance in haploid ES cells is unclear, so all of the haploid ES cells have to be enriched by FACS. Determining the very factors driving diploidization of haploid ES cells is critical for us to find proper chemical compounds that may maintain haploidy in routine culture instead of the Hoechst 33342 staining and FACS method that may compromise the developmental efficiency of haploid ES cells.

Moreover, deriving haploid cells in other species and other cell types is also essential. The generation of monkey phES cells suggests that haploid ES cells are also tolerated in primates, and thus it is valuable to derive human haploid ES cells in the future. Also, although monkey haploid ES cells should be useful in primate gene function research and recessive phenotype analysis, whether they can function like gametes as mouse haploid ES cells is unknown. Addressing this problem may provide insight into the assisted reproduction technology in humans through haploid ES cells.

Finally, whether differentiated haploid cells could be obtained and maintained is also an interesting point. We need to determine in the future whether the haploid status could be established in a somatic state rather than just in a pluripotent state.

## Abbreviations

ahES: androgenetic haploid embryonic stem; ES: Embryonic stem; FACS: Fluorescence-activated cell sorting; ICAI: Intracytoplasmic androgenetic haploid embryonic stem cell injection; phES: parthenogenetic haploid embryonic stem.

## Competing interests

The authors declare that they have no competing interests.
